# Medical students’ learning orientation regarding interracial interactions affects preparedness to care for minority patients: a report from Medical Student CHANGES

**DOI:** 10.1186/s12909-016-0769-z

**Published:** 2016-09-29

**Authors:** Diana J. Burgess, Sara E. Burke, Brooke A. Cunningham, John F. Dovidio, Rachel R. Hardeman, Yuefeng Hou, David B. Nelson, Sylvia P. Perry, Sean M. Phelan, Mark W. Yeazel, Michelle van Ryn

**Affiliations:** 1 Center for Chronic Disease Outcomes Research (a VA HSR & D Center of Excellence), Veterans Affairs Medical Center, 1 Veterans Drive (152/2E), Minneapolis, MN USA; 2 Department of Medicine, University of Minnesota, Minneapolis, MN USA; 3 Department of Psychology, Yale University, New Haven, CT USA; 4 Department of Family Medicine and Community Health, University of Minnesota, Twin Cities, Minneapolis, MN USA; 5 Division of Health Services Research, Policy & Administration, School of Public Health, University of Minnesota, Twin Cities, Minneapolis, MN USA; 6 3M Health Information Systems Division, Salt Lake City, USA; 7 Department of Psychology, Northwestern University, Evanston, IL USA; 8 Division of Health Care Policy and Research, Department of Health Sciences Research, Mayo Clinic, Rochester, MN USA

**Keywords:** Disparities, Medical education, Physician-patient relations, Attitude of health personnel

## Abstract

**Background:**

There is a paucity of evidence on how to train medical students to provide equitable, high quality care to racial and ethnic minority patients. We test the hypothesis that medical schools’ ability to foster a learning orientation toward interracial interactions (i.e., that students can improve their ability to successfully interact with people of another race and learn from their mistakes), will contribute to white medical students’ readiness to care for racial minority patients. We then test the hypothesis that white medical students who perceive their medical school environment as supporting a learning orientation will benefit more from disparities training.

**Methods:**

Prospective observational study involving web-based questionnaires administered during first (2010) and last (2014) semesters of medical school to 2394 white medical students from a stratified, random sample of 49 U.S. medical schools. Analysis used data from students’ last semester to build mixed effects hierarchical models in order to assess the effects of medical school interracial learning orientation, calculated at both the school and individual (student) level, on key dependent measures.

**Results:**

School differences in learning orientation explained part of the school difference in readiness to care for minority patients. However, individual differences in learning orientation accounted for individual differences in readiness, even after controlling for school-level learning orientation. Individual differences in learning orientation significantly moderated the effect of disparities training on white students’ readiness to care for minority patients. Specifically, white medical students who perceived a high level of learning orientation in their medical schools regarding interracial interactions benefited more from training to address disparities.

**Conclusions:**

Coursework aimed at reducing healthcare disparities and improving the care of racial minority patients was only effective when white medical students perceived their school as having a learning orientation toward interracial interactions. Results suggest that medical school faculty should present interracial encounters as opportunities to practice skills shown to reduce bias, and faculty and students should be encouraged to learn from one another about mistakes in interracial encounters. Future research should explore aspects of the medical school environment that contribute to an interracial learning orientation.

**Electronic supplementary material:**

The online version of this article (doi:10.1186/s12909-016-0769-z) contains supplementary material, which is available to authorized users.

## Background

Disparities between the quality of care received by white Americans and racial minorities have been documented extensively across a wide range of clinical areas and service types [1]. There is mounting evidence that physicians contribute, often unintentionally, to racial healthcare disparities through various processes, including poorer quality communication with racial minority patients and racial/ethnic biases in clinical decision-making [2–4]. As the U.S. becomes more racially and ethnically diverse, it is imperative that future physicians receive the training necessary to help reduce healthcare disparities.

Although there is now widespread endorsement from professional organizations of the need to prepare medical students to provide equitable, high quality care to racial and ethnic minority patients, there is a paucity of evidence on how to do so effectively. An important, yet overlooked, aspect of disparities-reduction training programs involves addressing how white medical students and physicians’ anxiety and uncertainty about interracial interactions can adversely affect the care of minority patients. Specifically, research has shown that whites generally find interactions with Blacks challenging both emotionally (e.g., anxiety producing) [5] and cognitively (e.g., resource depleting) [6]. These effects could impair the quality of care that white physicians give to racial minority patients, potentially impairing communication and increasing the likelihood that unconscious biases will influence clinical decision-making [7, 8]. Moreover, whites’ anxiety can be perceived as evidence of prejudice by members of minority groups [9, 10].

### The role of learning versus performance orientation on interracial interactions

Recent research has drawn upon the extensive literature on achievement motivation to understand how to improve whites’ motivation and ability to engage effectively in interracial interactions and reduce their concerns about being perceived as prejudiced [11–14]. Theories about learning distinguish between *learning goals*, in which people view challenging situations as opportunities to improve their skills and learn from their mistakes, and *performance goals*, in which the focus is on how one is perceived and evaluated [15]. Whereas learning goals lead to intrinsic motivation and persistence in the face of failure, performance goals lead to worries about making mistakes, greater anxiety, avoiding situations in which one might fail and decreases in intrinsic motivation [15].

Studies have found that having a learning orientation toward interracial interactions is associated with attitudes and behaviors conducive to positive interracial interactions. In two studies, interracial learning orientation was associated with greater comfort and interest in interracial contact among whites [12]. In another study, whites who were instructed to learn from an interracial interaction sat physically closer to their black partner than those who did not receive such instructions [16].

Additional support for the idea that learning orientation may help whites’ ability to interact with non-whites comes from several studies, examining the effects of a *growth mindset* about racial bias (i.e., that racial bias is changeable) versus a *fixed mindset* (that racial bias is not changeable). Studies have found that growth mindsets are more likely to engender learning goals, in contrast to fixed mindsets that are more likely to engender performance goals [14, 17]. In a series of eight studies by Carr et al. [11], majority group members who held (or were induced to hold) a growth mindset about prejudice were less interested in interracial interactions and prejudice reduction activities and were less comfortable and more anxious in interracial interactions than those who held (or were induced to hold) a fixed mindset [11]. Similarly, in a series of four studies by Neel and Shapiro [14], whites who believed or were induced to believe that racial bias is malleable were more likely to engage in learning-orientated strategies for interracial interactions (e.g., perspective-taking, getting feedback) compared to performance-oriented strategies (e.g. overcompensation, avoidance) than whites who believed or were induced to believe that bias was fixed.

There are several ways that medical school environments that foster a learning orientation regarding interracial interactions might improve white students’ ability to care for racial minority patients. Medical schools that promote a learning education may lead to a greater use of learning strategies that lead to improved communication skills (e.g., seeking out feedback and opportunities to improve one’s ability to interact effectively with members of racial and ethnic minority groups, perspective-taking and empathy) and decrease the use of performance strategies (e.g., strategic color blindness) and avoidance [14]. Interracial learning orientation should also increase the effectiveness of coursework related to the care of minority patients, as it is linked to a greater desire to engage in learning activities designed to promote racial diversity and reduce prejudice and to increased willingness to examine one’s own biases [12].

### The current study

The objective of the current study was to examine the effect of medical school interracial learning orientation at both the school and individual level, on white students’ readiness to provide equitable care for racial minority patients, using data from a national, longitudinal study of medical students, CHANGES. Perception of medical school learning orientation may vary from individual to individual within a medical school, even while the school exerts a common influence. It is reasonable to believe that (1) interracial learning orientation may differ among medical schools and this difference could contribute to school variance in readiness of white medical students to provide equitable care to racial minority patients in their final semester. Nonetheless, (2) it is likely that individual differences in perceived learning orientation in a medical school will exert a stronger influence on students than school-level differences.

We hypothesize that, among white students, school level interracial learning orientation will be positively associated with school level readiness to treat racial minority patients, as measured by self-assessments of (1a) self-efficacy in caring for a patient who is a member of a racial or ethnic minority, (1b) skill at developing positive relationship with racial minority patients, and (1c) interest in working with patients from a racial or ethnic minority group. Second, at the individual level, we hypothesize that white students’ perceptions of their medical school environment as supporting an interracial learning orientation will be positively associated with their readiness in caring for racial minority patients. Third, we explore whether individual level differences in perceived medical school learning orientation will moderate the effect of disparities training on white students’ readiness to care for minority patients. We focus on individual-level differences in learning orientation as a potential moderator, as we anticipate a much narrower range of perceived learning orientation among medical schools as compared to the amount of variance in students’ perceived learning orientation in a particular medical school, and expect it will be individual-level differences that will primarily affect their learning trajectory.

## Method

### Sample

Data are from the study of CHANGES Year 4 collection with medical students who entered medical schools in the fall of 2010 (i.e., Year 1). The purpose of the study (CHANGES) was to examine the impact of medical school factors on factors related to medical students’ clinical judgments and decisions related to patient race, between their first and last year of medical school. Forty-nine medical schools were randomly picked from strata of public and private schools in six regions of the United States. We ascertained students using three approaches: (1) emails of interested participants based on their responses to a question included as part of the Association of American Medical Colleges (AAMC) Matriculating Student Questionnaire; (2) a commercially available list of first year medical students; and (3) snowball sampling, through students who agreed to participate. Ascertained students were invited to participate in the study by postal mail and email. Each participant provided informed consent through an online written consent form. Study participants completed an online survey during their first semester of medical school between October 2010 and January 2011 (Year 1) and during their last semester of medical school between 2010 and 2014 (Year 4). See the following publications for additional description of the methods [18, 19]. For the purposes of the present examination, we included only participants who self-identified as white, did not identify with other racial/ethnic groups (e.g., were not biracial), who responded to the Year 4 measures, and were in their fourth year of medical school when the follow-up survey was administered (*N =* 2394).

### Measures

To see how referenced survey questions were displayed to participants, see Additional file 1.

### Measure of Medical School Learning Orientation towards Interracial Interactions

Our interracial learning orientation measure was derived from the mean of two items on the Year 4 survey, adapted from Migacheva and Tropp [12]: “Students in this medical school have the opportunity to learn how to interact more effectively with members of another race,” “Students in this medical school are encouraged to learn from their mistakes in interacting with members of another race.” Response options ranged from 1 “Strongly Disagree” to 7 “Strongly Agree” with higher scores indicating a greater learning orientation (Cronbach’s alpha = .783) [12].

### Coursework aimed at reducing disparities

We created a measure assessing hours of disparities-related training by averaging three items assessing the amount of training about (1) racial disparities in health care; (2) cultural customs that might affect clinical care, and (3) unintended racial bias (Range: 0–50 h, Cronbach’s alpha = .879). We selected these three items as the most relevant to disparities because exploratory factor analysis identified them as more closely related to each other than to our seven other non-disparities-related training items.

**Covariates** included gender, age, percentage of white students, region, whether the school was public/not public, and parental education (a proxy for socioeconomic status). Additional details about these measures are reported in [18, 19].

### Main Outcome Variables

**Readiness to Care for Minority Patients (1a through 1c)** was assessed using the following questions in the Year 4 survey. Q1a. We measured self-efficacy by asking students, “How prepared are you… to handle a patient who is a member of a racial or ethnic minority?” Response options ranged from 1 “Very Unprepared” to 5 “Very Well Prepared”. Q1b. We measured skills by asking students, “How skilled are you … in developing a positive relationship with racial minority patients.” Q1c. We measured interest by interest in working with minority students by asking students, “How interested are you in working with patients from a racial/ethnic group other than your own?” Response options ranged from 1 “Not at all interested” to 5 “Extremely interested.”

### Analyses

First Krippendorff’s alpha was used to test agreement among students, for calculating their medical schools’ learning orientation. The alpha of 0.76 was high enough to support the agreement among students within the same medical school. Therefore, we calculated the school-level learning orientation by taking the average of individual learning orientation scores within each medical school. We then employed a two-level mixed-effect hierarchical model to examine preconditions for the three hypotheses. The first level model (individual) used Readiness as the dependent variable and hours of disparity coursework (Hours) as the independent variable, with a random effect at the second level or school level model in both intercept and slope to assess the variance of Readiness and of effectiveness of coursework among medical schools. In order to test the first hypothesis, we added school level learning orientation as the main predictor along with the other school level covariates (i.e., percentage of whites in the medical school, public/private, region) in explaining the variance of intercept and with the individual level covariates (i.e., age, sex, parents highest education) at the first level model, when the variance in intercept was proved to be significant. For the second hypothesis, we built a second model by adding individual learning orientation at the individual level equation as a predictor to the previous model when the variance of slope among schools was proved not significant. The third hypothesis was tested by a third model with an interaction term (Learning Orientation X Hours) added to the individual level of the second model, wherein both Learning Orientation and Hours have been mean-centered to avoid multicollinearity problems.

## Results

Sample characteristics are presented in Table 1.

**Table 1:** Characteristics of the study sample

	Students (*N =* 2394)	Schools (*N =* 49)
Gender		
Female	1151 (48.1)	
Male	1243 (51.9)	
Region		
Northeast		12 (24.5 %)
Southeast		12 (24.5 %)
Central South		8 (16.3 %)
Midwest		11 (22.4 %)
Southwest		4 (8.2 %)
Northwest		2 (4.1 %)
Public Medical School		
Yes		31 (63.3)
No		18 (36.7)
Highest education of parents		
Less than Bachelor’s Degree	305 (12.7)	
Bachelor’s Degree	575 (24.0)	
Graduate Degree	1511 (63.1)	
Age^a^	24.0 (2.63)	
Percentage white students in school^a^		66.5 % (9.4)

*Note.* Unless otherwise indicated each cell reports the *N* (%)

^a^Cell reports the mean (SD)

### Primary analysis

Table 2 presents the descriptive statistics of self-efficacy, skills, interest, hours of disparity coursework, school learning orientation, and student learning orientation. As hypothesized, the range of school learning orientation (4–6) is much smaller than student learning orientation (1–7); in addition, the variance in intercept across medical schools is significant (0.014, *p =* .005) but the variance in slope across schools is not significant (0.00004, *p =* .387). Before fitting the three models, multicollinearity tests were conducted using Variance Inflation Factors (VIF) because both school level and student level learning orientation were used in the same model (*r* = .33), and no multicollinearity problem was detected.

**Table 2:** Descriptive statistics of self-efficacy, skills, interest, hours of disparity coursework, school learning orientation, and student learning orientation

	n	Max	Min	Mean	Std.
Self-efficacy	2366	5.00	1.00	4.29	0.71
Skills	2360	5.00	1.00	3.94	0.80
Interest	2359	5.00	1.00	3.70	0.97
Hours of disparity coursework	2337	50.00	0.00	11.68	9.45
School learning orientation	49	6.03	4.28	5.33	0.39
Student learning orientation	2350	7.00	1.00	5.42	1.31

### Model Test Results

Table 3 presents the results of the hierarchical models. The first hypothesis was that, among white students, school level interracial learning orientation would be positively associated with school level readiness to treat racial minority patients. This hypothesis was supported for Skill (β = 0.012, *p <* .001) and Self-Efficacy (β = 0.007, p.001), but not for Interest (β =−0.033, *p =* .633). This suggested that school level learning orientation made a difference in school level skills and self-efficacy in white students’ readiness to care for racial minority patients, but did not have an influence on their interest in providing such care.

**Table 3:** Effects of learning orientation and hours of disparities-related coursework on skill, self-efficacy, and interest at working with racial minority patients

	Skill	Self-efficacy	Interest
Hypothesis 1 learning			
School learning orientation	0.171 (0.052) *p =* .002	0.259 (0.053) *p <* .001	−0.033 (0.069) *p =* .633
Disparities coursework learning	0.012 (0.002) *p <* .001	0.007 (0.002) *p <* .001	0.004 (0.002) *p =* .092
Hypothesis 2			
School learning orientation	0.026 (0.051) *p =* .668	0.152 (0.052) *p =* .006	−0.119 (0.066) *p =* .078
Disparities coursework	0.008 (0.002) *p <* .001	0.004 (0.002) *p =* .037	0.001 (0.002) *p =* .688
Student learning orientation	0.156 (0.015) *p <* .001	0.115 (0.013) *p <* .001	0.096 (0.021) *p <* .001
Hypothesis 3			
School learning orientation	0.030 (0.050) *p =* .56	0.157 (0.051) *p =* .004	−0.111 (0.066) *p =* .097
Disparities coursework	0.007 (0.002) *p =* .001	0.003 (0.002) *p =* .213	−0.001 (0.002) *p =* .645
Student learning orientation	0.160 (0.015) *p <* .001	0.119 (0.013) *p <* .001	0.104 (0.020) *p <* .001
Student learning orientation X disparities coursework	0.003 (0.001) *p =* .051	0.004 (0.001) *p =* .012	0.006 (0.002) *p =* .004

*Note.* This table summarizes a number of hierarchical linear models. Each cell reports an unstandardized slope (*b*) for a variable of interest, the standard error of that slope (in parentheses), and the *p*-value. All models include a random intercept by school and include region of the country (Northeast, Southeast, Central South, Midwest, Southwest, Northeast), public/private and percentage of white students (based on data collected by the AAMC in 2010) as school level covariates, and student age, gender, and the highest education level of either parent as student-level covariates

The second hypothesis, that white students’ perceptions of their medical school environment as supporting an interracial learning orientation would be positively associated with their readiness in caring for racial minority patients, was confirmed for all three readiness measures: Skill (β = 0.156, *p <* .001), Self-Efficacy (β = 0.115, *p <* .001), and Interest (β = 0.096, *p <* .001). This suggests that students’ perceptions of medical school learning orientation was associated with their readiness to provide care for racial minority patients, even after accounting for variation in learning orientation at the school level.

**Fig. 1:**
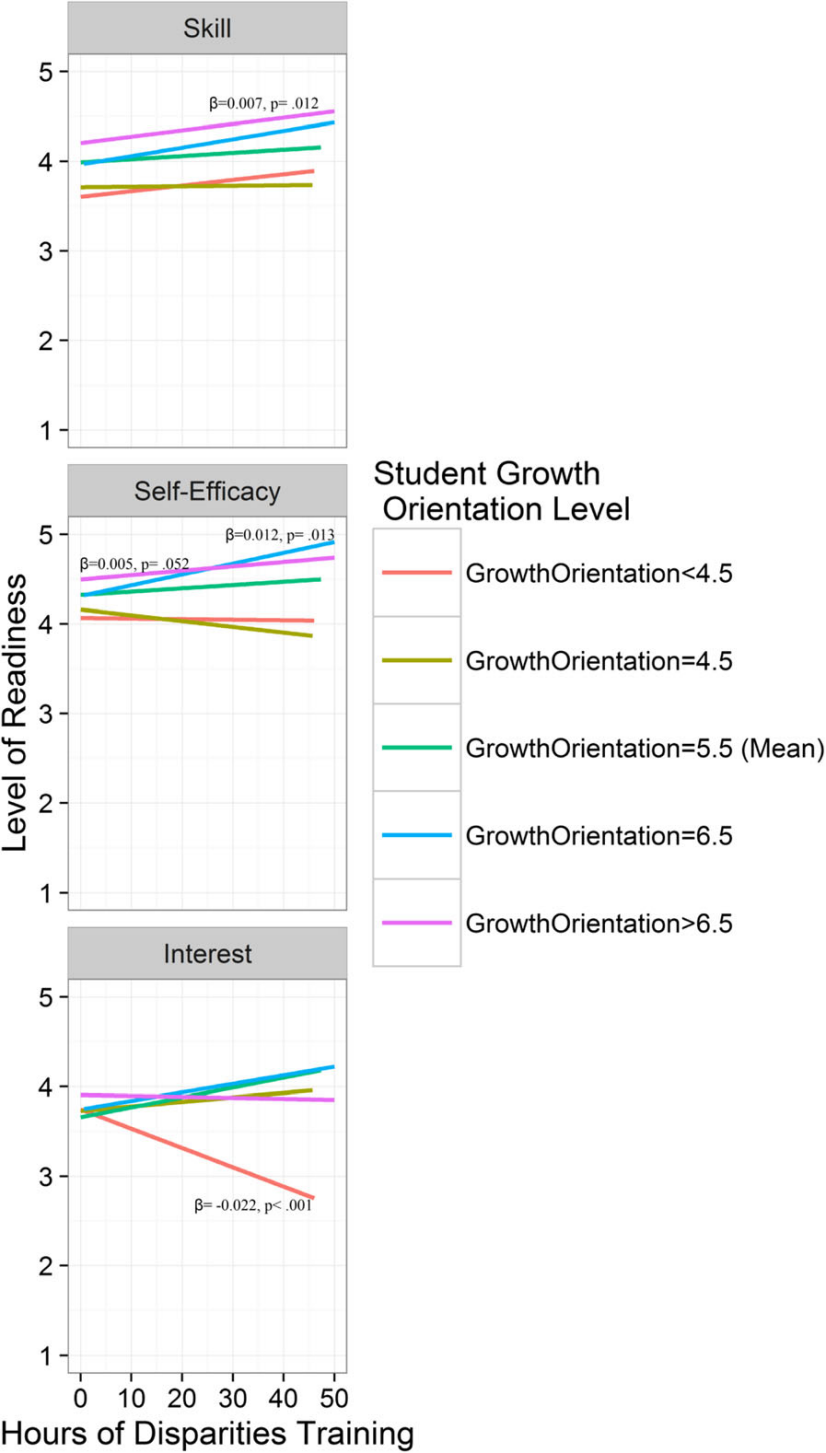
Effect of growth orientation and hours of disparities training on perceived skill developing a positive relationship with racial minority patients, self-efficacy in working with racial minority patients, and interest in working with racial minority patients

In our third series of analyses, we explored whether perceptions of medical school learning orientation (at the individual level) would moderate the effect of disparities training on white students’ readiness to care for minority patients. The individual level interaction term (student learning orientation X disparities coursework) was significantly associated with white students’ self-efficacy (β = 0.004, *p =* .012) and interest (β = 0.006, *p =* .004) and marginally significantly associated with skills (β = 0.003, *p =* .051). The effect sizes and directions of the student learning orientation as a moderator for disparities coursework have been illustrated in Fig. 1, in which only the significant or marginally significant slopes have been marked. Hours of disparities coursework were shown to be positively associated with Skill and Self-Efficacy only at high level of student learning orientation (i.e., > = 6.5); Hours could even be negatively correlated with Interest at a very low level of student learning orientation (i.e., <4.5).

## Discussion

This study adds to the nascent empirical literature on how to best prepare future physicians to provide equitable and high quality care to racial and ethnic minority patients. School level differences in promoting a learning orientation toward interracial interactions were associated with school level differences in students’ skills and self-efficacy for caring for racial minority patients (although school level differences were not associated with interest in providing such care). As expected, individual differences in learning orientation were more important than school level differences in affecting readiness to care for minority patients: they were associated with skills, self-efficacy and interest, even after accounting school level differences. Additionally, the extent to which formal coursework on health disparities/minority health was associated with greater self-efficacy, skill and interest in working with minority patients depended on whether students perceived their medical school environment as fostering an interracial learning orientation (after accounting for school level differences). This suggests that fostering an interracial learning orientation within the context of medical school can protect against unintended consequences of disparities programs and enhance the effectiveness of such programs.

There are several limitations to this study. First, we did not directly measure medical students’ behavior and skills, relying instead on self-assessment. However, prior research has shown that physicians’ self-assessments of cultural competency and interracial anxiety are positively associated with better outcomes and levels of satisfaction among minority patients [20–22]. A second limitation is that perceptions of school interracial learning orientation and self-assessed preparedness, skills, and interest in working with minority patients were only measured at Year 4, precluding our ability to make inferences about whether interracial learning orientation precedes these other variables. It is possible, for instance, that students who entered medical school with relatively high levels of efficacy, skill and interest in working with minority populations, self-selected into medical schools that were strong in this area.

Several practical implications flow from this work. First, our study suggests that the formal and informal curriculum should be aligned to foster an interracial learning orientation [8]. Faculty should present interracial encounters as opportunities to practice various skills shown to reduce bias and/or the impact of bias [8, 23]. Also faculty and students should be encouraged to learn from one another about mistakes in inter-racial encounters. Medical schools already aim to prepare students to address and psychologically cope with medical error. Explicitly discussing mistakes in interracial encounters could reduce interracial anxiety and ultimately improve interracial doctor-patient relationships. Requiring faculty to participate, both with students and in faculty-only trainings, will bring what is often hidden (in the “hidden curriculum” [24] or buried due to discomfort) to the forefront.

Similarly cultural competency or related programs can both make students’ aware of the ways in which bias leads to mistakes in medical decision making, and offer strategies to increase bias awareness and reduce racial bias. This may produce iterative change as greater belief in malleability (a growth mindset) is associated with an individual’s learning orientation. This is particularly important in light of prior work showing that healthcare providers’ beliefs regarding the malleability of racial bias declined after participation in a program designed to increase awareness about implicit bias in healthcare [25]. Programs might include components modeled on prior interventions that have demonstrated sustained effects in changing students’ beliefs about the malleability of intelligence [26, 27]. It would also be useful to frame conversations about racial bias in healthcare in terms of “biased behaviors,” which implies the potential for change rather than “racially biased physicians” which implies that racial bias is fixed [8].

## Conclusions

Coursework aimed at reducing healthcare disparities and improving the care of racial minority patients was only effective when white medical students perceived their school as having a learning orientation toward interracial interactions. This work suggests a missing piece to the disparities education puzzle. Disparities programs best achieve their intended goals to produce greater self-efficacy and skills at caring for racial and ethnic minority patients when they are instituted in schools with a high learning orientation. Future research should explore other aspects of the medical school environment that contribute to an interracial learning orientation.

## Additional file

Additional file 1: Survey Questions: Survey Questions: Measures and main outcomes survey questions. (DOCX 163 kb)

## Abbreviations

AAMC: Association of American medical colleges
CHANGES: Cognitive Habits and Growth Evaluation Study
HSR&D: Health science
U.S.: United States
VA: Veterans affairs
VIF: Variance inflation factors
