# Supplementary material for: Medical students’ learning orientation regarding interracial interactions affects preparedness to care for minority patients: a report from Medical Student CHANGES
Source: BMC Med Educ. 2016 Sep 29;16:254. doi: 10.1186/s12909-016-0769-z (PMC5041316; doi:10.1186/s12909-016-0769-z)

**Supplementary Material – Survey Questions**

**Main Outcome Variables**

**Q1a**


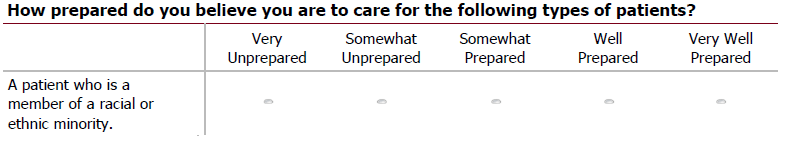


**Q1b**


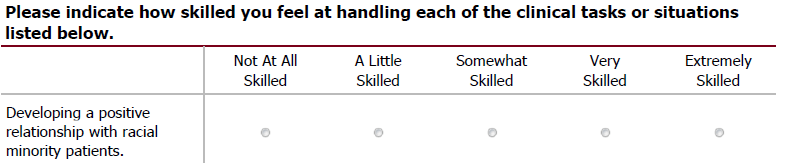


**Q1c**


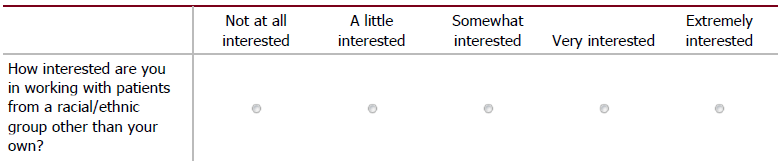


**Measure of Medical School Learning Orientation towards Interracial Interactions.**


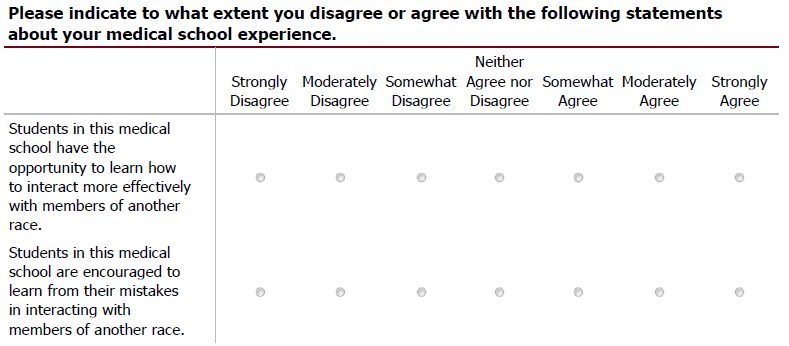


**Coursework aimed at reducing disparities**.


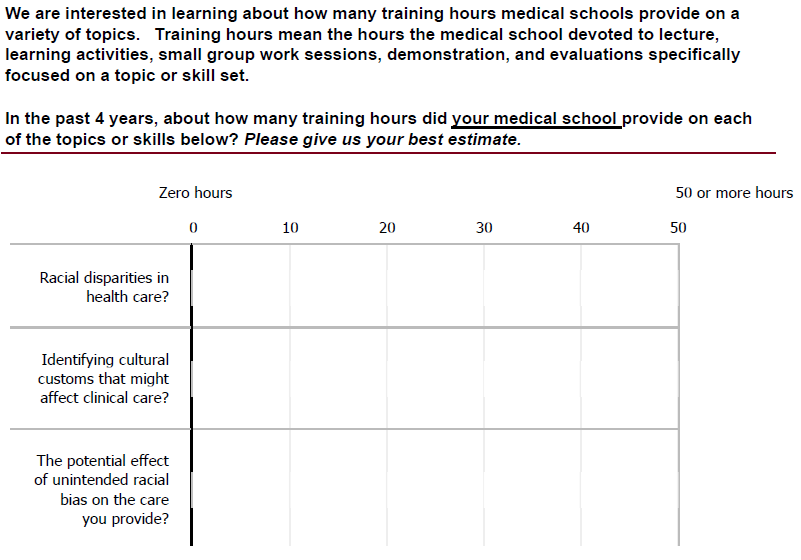

Supplement: Additional file 1: — Survey Questions: Survey Questions: Measures and main outcomes survey questions. (DOCX 163 kb) [file 12909_2016_769_MOESM1_ESM.docx]
